# Re-Excision After Positive Margins in Breast-Conserving Surgery: Can a Risk-Based Strategy Avoid Unnecessary Surgery?

**DOI:** 10.3390/jcm14165839

**Published:** 2025-08-18

**Authors:** Sabatino D’Archi, Beatrice Carnassale, Cristina Accetta, Flavia De Lauretis, Enrico Di Guglielmo, Alba Di Leone, Antonio Franco, Federica Gagliardi, Stefano Magno, Francesca Moschella, Maria Natale, Eleonora Petrazzuolo, Alejandro Martin Sanchez, Lorenzo Scardina, Marta Silenzi, Gianluca Franceschini

**Affiliations:** 1 Multidisciplinary Breast Centre, Dipartimento Scienze della Salute della Donna e del Bambino e di Sanità Pubblica, Fondazione Policlinico Universitario A. Gemelli IRCCS, 00168 Rome, Italycristina.accetta@policlinicogemelli.it (C.A.); flavia.delauretis@guest.policlinicogemelli.it (F.D.L.); enricodiguglielmo@yahoo.com (E.D.G.); stefano.magno@policlinicogemelli.it (S.M.); gianluca.franceschini@policlinicogemelli.it (G.F.); 2 Dipartimento di Scienze Mediche e Chirurgiche, Università Cattolica del Sacro Cuore, 00168 Rome, Italy

**Keywords:** breast cancer, surgical treatment re-excision, breast-conserving surgery, positive margins, residual tumour

## Abstract

**Background:** Re-excision after breast-conserving surgery (BCS) is routinely recommended when positive margins are found. However, secondary surgery often reveals no residual disease, exposing patients to unnecessary interventions that compromise cosmetic outcomes, increase costs, and reduce quality of life. This study investigates clinicopathological predictors of a residual tumour to identify low-risk patients who may safely avoid re-excision. **Methods:** We conducted a retrospective cohort study of 135 patients who underwent reoperation for positive margins following BCS at the Breast Unit of Fondazione Policlinico Universitario A. Gemelli IRCCS in Rome, between 2019 and 2024. Data on patient demographics, tumour characteristics, and histopathological findings were analyzed using univariate and multivariate models to identify predictors of residual disease. **Results:** A residual tumour was detected in 66 of 135 patients (48.9%). In the remaining 69 cases (51.1%), no residual disease was found, indicating that re-excision may have been unnecessary. Multifocality (*p* < 0.01), lymphovascular invasion (LVI) (*p* < 0.05), and involvement of ≥2 margins (*p* < 0.05) were independently associated with the residual tumour. Patients with unifocal disease, absence of LVI, and a single positive margin had a significantly lower risk of residual disease. **Conclusions:** Over half of re-excisions performed for positive margins may be avoidable. A risk-adapted approach incorporating tumour focality, LVI status, and margin involvement can help identify patients for whom secondary surgery may offer limited benefits. These findings support a more individualized strategy to margin management in BCS aimed at reducing overtreatment without compromising oncologic safety.

## 1. Introduction

Breast-conserving surgery (BCS) has become the gold standard in the treatment of early-stage breast cancer, offering survival rates comparable to a mastectomy while preserving breast appearance and improving patient quality of life [1]. To achieve oncologic outcomes equivalent to a mastectomy, BCS must be accompanied by appropriate adjuvant therapies and meticulous surgical technique, particularly in margin management [2,3].

Traditionally, a clear rim of uninvolved tissue between the tumour and inked surgical margins was required to ensure an adequate resection. However, current guidelines define a positive margin as the presence of tumor cells directly at the inked edge (‘ink on tumor’) [4]. Positive margins are associated with a higher likelihood of residual disease and approximately double the risk of local recurrence, even when favourable tumor biology or adjuvant therapy is present [5].

Meta-analyses estimate that 8–10% of BCS cases result in positive margins. This rate is now a key quality indicator in breast cancer surgery. According to EUSOMA standards, high-performing centres should aim for a positive margin rate below 10% [6].

Guidelines recommend a second surgery, either re-excision or completion mastectomy, in cases of positive margins. However, this comes at a cost: secondary surgery can negatively affect cosmetic results, delay systemic therapies, increase perioperative risk, and significantly impact patients’ psychological well-being. Moreover, growing interest in surgical de-escalation and precision oncology has prompted a critical re-evaluation of whether re-excision is always necessary.

Several retrospective studies have shown that over 50% of re-excised specimens reveal no residual tumour, suggesting that a substantial proportion of reoperations may be oncologically unnecessary [7]. Despite this, reliable predictors of residual disease remain underexplored and inconsistently reported.

This study aims to assess the incidence of residual tumours following a margin re-excision and to identify clinicopathological factors predictive of residual disease. The ultimate goal is to define a subset of patients at low risk, in whom reoperation may be safely avoided.

## 2. Materials and Methods

### 2.1. Study Design and Patients

This retrospective cohort study included 135 female patients who underwent BCS followed by a second surgical procedure (re-excision or completion mastectomy) due to positive margins at the Breast Unit of the Fondazione Policlinico Universitario A. Gemelli IRCCS in Rome, Italy, between January 2019 and December 2024.

Patients were eligible if they

- Had histologically confirmed invasive breast cancer;
- Underwent BCS as a primary surgical treatment;
- Required a second operation due to positive surgical margins on final pathology.

Exclusion criteria were

- Reoperations performed for postoperative complications, recurrence, or patient preference unrelated to margin status;
- Missing or incomplete histopathological or clinical data.

### 2.2. Margin Definition and Surgical Technique

Positive margins for invasive carcinoma were defined according to current guidelines as the presence of tumour cells at the inked surface of the specimen (‘ink on tumor’).

Preoperative localization of non-palpable lesions was performed using ultrasound- or mammography-guided skin marking. Surgical margins were assessed intraoperatively in selected cases using specimen radiography or gross pathological examination. All surgical decisions were made by a multidisciplinary team (MDT) including breast surgeons, radiologists, pathologists, oncologists, and radiation oncologists, and were shared with the patient.

To identify the surgical margins in both standard and oncoplastic procedures, our shared institutional protocol includes the placement of metallic clips at the resected margins of the tumour cavity. This approach aims to facilitate potential margin re-shaving or the delivery of an adjuvant radiotherapy boost, if needed.

Adjuvant radiotherapy (including boost) was administered according to institutional protocols and MDT decisions, considering patient age, margin status, and tumour biology.

### 2.3. Data Collection and Variables

Demographic, clinical, imaging, surgical, and histopathologic data were collected from electronic medical records. The following variables were analyzed:

- Patient-related factors: Age at diagnosis (≤50 vs. >50 years), breast density (BI-RADS classification), preoperative MRI usage, and presence of microcalcifications.
- Tumour characteristics: Size (TNM classification), histological type and grade (modified Bloom–Richardson), immunophenotype (ER, PR, HER2, and Ki-67), lymph node status, presence of an extensive intraductal component (EIC), lymphovascular invasion (LVI), and multifocality.
- Margin-related variables: Number of positive margins (1 vs. ≥2), margin width (if available), and type of second surgery (re-excision vs. mastectomy).
- Residual disease: Defined as the presence of invasive carcinoma and/or DCIS in the re-excision specimen.

In multifocal tumours, the largest lesion was considered for size and histopathological assessment. The EIC was defined as present when >25% of the tumour contained intraductal components.

### 2.4. Statistical Analysis

Univariate analysis was performed using a Fisher’s exact test or Chi-square test, as appropriate, to evaluate associations between clinical and pathological variables and the presence of a residual tumour at re-excision.

Variables with *p* ≤ 0.05 in univariate analysis were entered into a multivariate logistic regression model using a backward stepwise method to identify independent predictors. Odds ratios (ORs) and 95% confidence intervals (CIs) were calculated.

All statistical analyses were conducted using IBM SPSS Statistics version 24.0 (IBM Corp., Armonk, NY, USA). A *p*-value ≤ 0.05 was considered statistically significant.

## 3. Results

Between January 2019 and December 2024, a total of 135 patients who underwent breast-conserving surgery (BCS) followed by a second surgical procedure for positive margins were included in the study. The mean patient age was 52.7 years (range: 28–85). Patient and tumour characteristics are summarized in Table 1.

**Figure 1:**
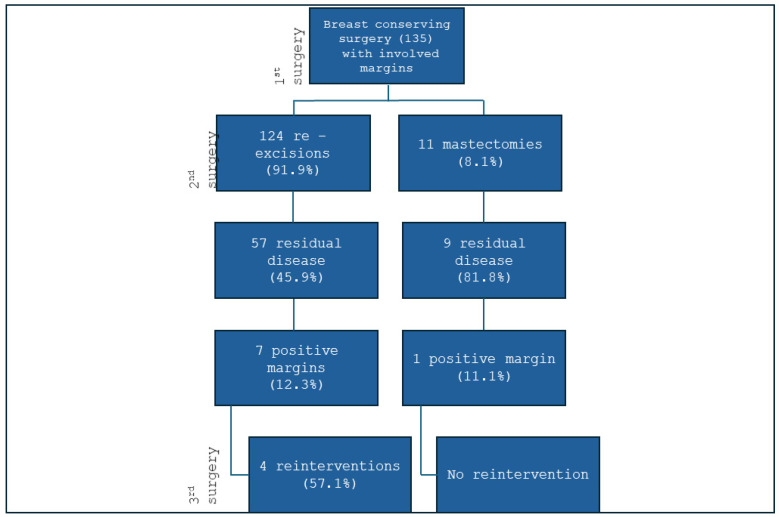
Surgical treatment of patients who underwent re-excision for positive margins.

Of the 135 patients who required reoperation, 124 (91.9%) underwent re-quadrantectomy, while 11 (8.1%) underwent a completion mastectomy. A residual tumour was identified in 66 patients, corresponding to an overall residual disease rate of 48.9% (Figure 1). This indicates that 51.1% of re-excision specimens showed no residual disease and were potentially unnecessary.

### 3.1. Univariate Analysis

A univariate logistic regression identified the following variables as significantly associated with the presence of a residual tumour in re-excision specimens:

- Multifocality: OR 3.06, 95% CI: 1.47–6.35, and *p* = 0.0027;
- LVI: OR 5.97, 95% CI: 2.10–17.02, and *p* < 0.001;
- Number of positive margins (≥2): OR 2.30, 95% CI: 1.48–3.59, and *p* < 0.001.

These associations were based on histopathologic findings from the initial conservative surgical specimens.

### 3.2. Multivariate Analysis

**Figure 2:**
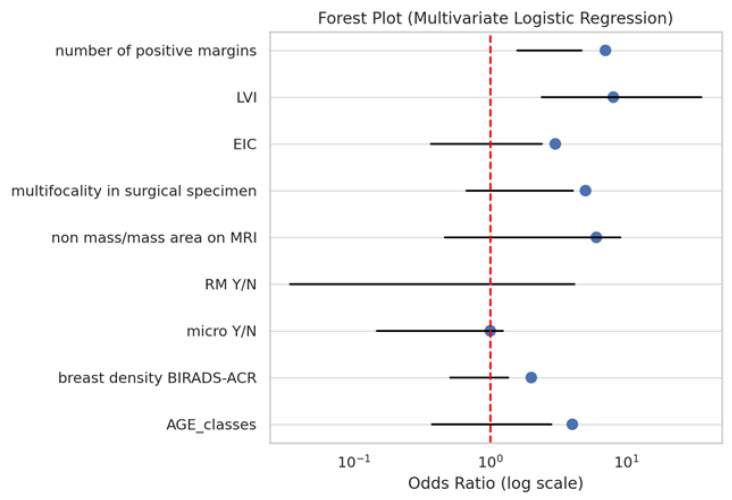
Forest plot from multivariate logistic regression for predictors of residual tumour.

In the multivariate logistic regression model, two variables remained independently associated with the presence of a residual tumour (Figure 2, Table 2):

- LVI: OR 9.21, 95% CI: 2.39–35.47, and *p* = 0.0013;
- Number of positive margins (≥2): OR 2.73, 95% CI: 1.58–4.69, and *p* < 0.001.

Although multifocality was significant in the univariate analysis, it did not retain independent significance in the multivariate model. This suggests potential collinearity or confounding, particularly with LVI, which may serve as a stronger marker of biological aggressiveness.

The multivariate model demonstrated a good discriminative performance with an area under the ROC curve (AUC) of 0.81, indicating a strong ability to distinguish between patients with and without a residual tumour.

## 4. Discussion

The rate of surgical re-excision for infiltrated margins varies in the literature from 10% to 57% [7], depending on the definition of the tumour margin width used. Margin status is considered the most important predictor of local recurrence, but the correct surgical resection margin remains a matter of debate. While a wide resection margin was deemed necessary in the past, today, the guidelines state that it is sufficient to have no ink on the tumour [4]. Several meta-analyses [5,8] have shown that while a higher recurrence rate occurs with ink on the tumour versus margins of at least 1 mm, widths greater than 1 mm offer no statistical benefit. The main conclusion is that there is no justification for margins > 1 mm in patients undergoing BCS [8]. Conversely, a recent meta-analysis by Bundred and colleagues [9] provided enough data to compare ‘no ink on tumour’ with 1 mm. They concluded that a minimum negative margin should be set at 1 mm instead of ‘no ink on tumour’ and recommended revising international guidelines accordingly. Additionally, a recent review [8] reinforced this viewpoint, suggesting that guidelines should redefine a negative margin as 1 mm instead of ‘no ink on tumour’ in the context of BCT. The broader implications of this change require thorough examination. The total number of patients affected may be considerable, especially in busy breast units, leading to more re-excision procedures that could strain healthcare systems and diminish patient quality of life. Additional surgeries introduce risks of complications, cosmetic issues, delays in adjuvant therapies, and increased costs. Moreover, 30–70% of patients undergoing re-excision for inadequate margins show no remaining disease [10]. These findings align with the current SSO-ASTRO-ASCO consensus guidelines, which emphasize the importance of minimizing unnecessary re-excisions without compromising oncologic outcomes [4,11]. The necessity for individualized re-excision strategies is also highlighted in more recent data, indicating that residual disease may still be present despite margin clearance, particularly in aggressive tumour subtypes [12,13].

In our study, 51.5% of the patients with positive surgical margins displayed no evident residual cancer during reoperation. This finding is congruent with data reported in most of the existing literature [14,15,16], although considerable variability exists [17,18,19]. Several factors may clarify the high absence rate of residual tumours in surgical reinterventions. Research indicates a significant occurrence of ‘false positives’ due to technical mistakes, such as ink penetrating through cracks in the surgical specimen, which can create a misleading impression of margin infiltration [20]. Other studies point to the ‘pancake phenomenon,’ where the specimen flattens between excision and evaluation, combined with the shrinking effect of formalin, which may result in the tumour appearing nearer to the excision margin than it truly is in vivo [21,22]. Another explanation involves how surgery impacts the remaining breast tissue, potentially lowering the likelihood of residual disease by destroying remaining tumour cells directly through diathermy or inducing an inflammatory reaction [23]. In some cases, the residual tumour may appear in the re-excision specimen but may not be detected in the reviewed slides, as it is widely acknowledged that examining the entire tissue is not practically possible in a sample [20]. Ultimately, from a surgical perspective, conducting a targeted re-excision of the infiltrated margin presents challenges, especially in recent years due to the growing prevalence of oncoplastic remodelling, which involves the extensive mobilization of glandular flaps [23,24].

In our study, over half of the re-excisions were deemed unnecessary given that no residual disease was identified. We examined various clinicopathologic factors to determine their effectiveness in identifying patients with positive or close margins who could safely avoid additional surgery.

In the present study, tumour multifocality emerged as a significant risk factor for residual disease following breast-conserving surgery in the univariate analysis. Specifically, 66% of patients with multifocal breast cancer were found to have residual disease, compared to only 38.8% of patients with unifocal tumours. These findings align with those reported by Sabel et al. [25], who identified multifocality as the sole variable significantly associated with residual disease in both univariate and multivariate analyses. In their cohort, over 50% of patients with multifocal tumours had residual disease upon re-excision, in contrast to fewer than 25% of patients with unifocal disease [25]. Further evidence supports these observations. Gurdal et al. reported that patients with multifocal tumours had a more than fivefold increased risk of residual disease at re-excision or mastectomy compared to those with unifocal tumours (OR = 5.2, 95% CI: 2.6–10.4) [7]. Additionally, a meta-analysis by Sun et al. demonstrated that patients with multifocal or multicentric breast cancer undergoing breast-conserving therapy had significantly higher rates of local recurrence compared to those with unifocal tumours [26]. Similarly, Saarela et al. found that multifocality was significantly associated with positive histologic margins and residual disease in re-excisions (*p* = 0.001), recommending routine re-excision in such cases [27]. Rath et al. also identified multifocality as an independent predictor of residual tumour in multivariate analysis (*p* = 0.002), alongside tumour size and presence of DCIS [28]. These findings support our data and suggest that tumour multifocality should be considered a strong indicator for the presence of residual disease, potentially guiding more aggressive surgical management.

In our study, the number of involved margins following BCS was significantly associated with residual disease upon re-excision in both univariate and multivariate analyses. Specifically, a residual tumour was detected in 84% of cases with three involved margins, 52.8% with two, and 26.5% with one.

These results are consistent with the prior literature highlighting the prognostic value of the extent of margin involvement. Simpson et al. evaluated 220 patients undergoing re-excision and found a clear linear trend between the number of involved margins and the likelihood of residual disease: patients with a single involved margin had a significantly lower residual tumour rate than those with two or more. Their multivariate analysis confirmed this trend as statistically significant (*p* < 0.001), supporting the number of involved margins as an independent predictor of residual disease [23]. Vos et al. analyzed 499 patients and stratified margin involvement into four categories: negative, close, focally positive (≤4 mm of tumour at the ink), and extensively positive (>4 mm). They found that residual disease was present in 50% of cases with focally positive margins and 70% of those with extensively positive margins. Although not reported per number of margins, the extent of involvement directly paralleled our stratification, indicating that a more extensive or multifocal margin positivity significantly raises the risk of residual disease [29]. Similarly, Patterson et al. conducted a large retrospective cohort study on 1001 patients with DCIS, reporting that both the number and type of involved margins were significantly associated with residual disease on re-excision. The statistical significance for a number of positive margins remained robust in multivariate analysis (*p* = 0.004), and the presence of multiple involved margins substantially increased the probability of residual disease [30]. In our analysis, we included only patients diagnosed with invasive breast carcinoma. However, data emerging from the literature suggest that a similar approach could also be applied to cases of DCIS.

In our study, the presence of LVI on the final histopathological examination was significantly associated with the presence of residual disease following a margin re-excision after breast-conserving surgery in both univariate and multivariate analyses. Specifically, 80.8% of patients with LVI had a residual tumour at reoperation, compared to only 41.3% of patients without LVI. This pronounced difference highlights the potential role of LVI as a reliable indicator of microscopic disease spread beyond the primary tumour bed.

Prior studies strongly support this observation. Alrahbi et al. reported that LVI was significantly associated with residual tumours after margin re-excision in breast-conserving surgery [31]. In their retrospective cohort of 720 patients, LVI, along with an extensive intraductal component and the involvement of more than one radial margin, emerged as a key predictor of residual disease. Similarly, Ozmen et al. found a statistically significant association between LVI and residual tumours in re-excision specimens (*p* = 0.029), reinforcing its role as a marker for occult microscopic spread [32]. Although not always statistically significant, other studies have consistently supported this association. For example, Atalay and Irkkan noted that LVI approached significance (*p* = 0.064) as a predictive factor for a residual tumour following re-excision in patients with positive margins [33]. Together, these findings align with our results and suggest that the presence of LVI should be considered a strong risk factor for residual disease, potentially guiding the decision to perform margin re-excision.

Several studies have demonstrated a correlation between a younger age and an increased risk of a residual tumour following surgical re-excision for positive margins [34,35]. Our findings indicate that age, categorized as below or above 50 years, did not reach statistical significance as a predictor of residual tumours.

Our results regarding age are consistent with those of Mendoza-Rojas et al., who found no significant association between age and residual tumours in their cohort of early-stage breast cancer patients undergoing re-excision [36]. However, other studies have reported that a younger age, particularly under 45 years, is an independent risk factor for residual disease following breast-conserving surgery [34,35]. These discrepancies may be attributed to variations in study populations, surgical techniques, and approaches for younger patients.

According to our dataset, HER2 3+ and Luminal B subtypes were more frequently observed among patients with a residual tumour, suggesting a possible trend that did not achieve statistical significance. This evidence is supported by findings from Atalay and Irkkan, who identified HER2 positivity as a significant predictor of residual tumours in re-excision specimens [36]. However, other studies, such as that by Haideri et al., have not found consistent associations between hormone receptor status or HER2 expression and residual tumours, suggesting that these factors may be influenced by additional variables such as tumour size or margin width [37].

### Limitations of the Study

This study has several limitations that should be acknowledged. First, the retrospective and single-centre nature of the analysis may introduce selection bias and limit the generalizability of our findings to other institutions with different surgical protocols and patient populations. Second, the relatively small sample size may have reduced the statistical power to detect subtle associations, especially in subgroup analyses. Moreover, certain potentially relevant factors—such as the exact width and anatomical location of positive margins, the type of oncoplastic technique used, and the extent of glandular remodelling—were not systematically captured or included in the multivariate model.

Another limitation of our study is the absence of systematic data collection on the use of a radiotherapy boost as an alternative to re-excision. Although some evidence suggests that high-dose boosts may provide adequate local control, particularly in patients with focally positive margins [38], our retrospective analysis did not consistently capture whether boost radiotherapy was employed as a compensatory strategy in these cases. This observation could serve as a valuable starting point for further and more in-depth investigations.

Furthermore, we did not investigate long-term oncological outcomes such as local recurrence or disease-free survival, which could reinforce the clinical impact of avoiding re-excision in low-risk patients. Although our findings highlight key clinicopathological predictors of residual tumours, these results should be interpreted within the context of these limitations.

Another limitation of our study is the exclusion of cases with a preoperative diagnosis of pure DCIS from the analysis. Our aim is therefore to investigate, in future studies, the correlation between residual disease and predictive prognostic factors in cases diagnosed with DCIS without preoperative evidence of an invasive component.

Importantly, these limitations also represent valuable opportunities for future research. Prospective, multicentre, and potentially randomized studies are needed to validate our proposed risk-adapted approach and to determine whether omission of re-excision in selected patients is both oncologically safe and beneficial in terms of quality of life, cosmesis, and resource utilization.

## 5. Conclusions

This study suggests that a significant proportion of re-excisions following breast-conserving surgery for positive margins may be oncologically unnecessary with over 50% of patients showing no residual tumour. These findings highlight the need for a more nuanced risk-adapted approach to margin management.

Our results identify LVI, tumour multifocality, and the number of involved margins as strong predictors of residual disease. In particular, LVI and multiple positive margins retained independent significance in a multivariate analysis. Conversely, patients with unifocal tumours, no LVI, and only one involved margin had a notably lower likelihood of residual disease.

However, these findings stem from a single-centre, retrospective cohort and should not be interpreted as a basis for immediate changes in clinical practice. Instead, our results serve as a proof-of-concept that supports the rationale for designing prospective, multicentre—and potentially randomized—studies aimed at validating a risk-adapted strategy for margin re-excision.

In the era of precision medicine, rather than adopting a uniform threshold for reoperation, surgical decisions should be guided by a combination of pathological and clinical risk factors. This tailored approach can reduce unnecessary procedures, preserve cosmetic outcomes, avoid treatment delays, and optimize resource use without compromising oncological safety.

## 6. Key Implications

- Routine re-excision after positive margins may lead to overtreatment in up to 50% of cases.
- LVI and multiple margin involvement are strong independent predictors of residual tumours.
- A risk-adapted approach to re-excision should be incorporated into multidisciplinary surgical decision-making.
- Clinical guidelines should evolve to consider tumour biology and patient-specific factors alongside margin status.

## Figures and Tables

**Table 1:** Factors associated with residual tumour after re-excision for positive margins.

FACTORS	NO RESIDUAL DISEASE (n = 69 51%)	RESIDUAL DISEASE (n = 66 49%)	*p* Value
Age classes			0.723
≥50 y.o: 47	25 (53.2%)	22 (46.8%)	
>50 y.o: 88	44 (50%)	44 (50%)	
Breast density (ACR-class)			0.473
a: 35	16 (45.7%)	19 (54.3%)	
b: 38	20 (52.6%)	18 (47.4%)	
c: 37	19 (51.4%)	18 (48.6%)	
d: 25	14 (56%)	11 (4%)	
Microcalcifications			0.282
Y: 47	27 (57.4%)	20 (42.6%)	
N: 88	42 (47.7%)	46 (52.3%)	
Preoperative MRI			0.871
Y: 38	19 (50%)	19 (50%)	
N: 97	50 (51.5%)	47 (48.5%)	
Mass/non-mass MRI evidence			0.446
Non mass: 21	13 (61.9%)	8 (38.1%)	
Mass: 17	6 (35.3%)	11 (64.7%)	
pT			0.727
1	51 (53.7%)	44 (46.3%)	
2	16 (42.1%)	22 (57.9%)	
3	1 (100%)	0 (0%)	
4	1 (100%)	0 (0%)	
pN			0.302
0	54 (51.9%)	50 (48.1%)	
1	11 (40.7%)	16 (59.3%)	
2	4 (100%)	0 (0%)	
Multifocality			**0.002**
Y: 50	17 (34%)	33 (66%)	
N: 85	52 (61.2%)	33 (38.8%)	
EIC			0.518
Y: 47	26 (55.3%)	21 (44.7%)	
N: 86	42 (48.8%)	44 (51.2%)	
Not reported	1 (50%)	1 (50%)	
LVI			**<0.001**
Y: 26	5 (19.2%)	21 (80.8%)	
N: 109	64 (58.7%)	45 (41.3%)	
Number of positive margins			**<0.001**
1: 49	36 (73.5%)	13 (26.5%)	
2: 53	25 (47.2%)	28 (52.8%)	
3: 25	4 (16%)	21 (84%)	
4: 8	4 (50%)	4 (50%)	
Histotype			0.419
CDI: 90	46 (51.1%)	54 (48.9%)	
CLI: 21	13 (61.9%)	8 (38.1%)	
Special type: 12	8 (66.7%)	4 (33.3%)	
Mesenchymal: 2	2 (100%)	0 (0%)	
Immunophenotype			0.894
LUM A: 60	33 (55%)	27 (45%)	
LUM B: 46	22 (47.8%)	24 (52.2%)	
HER2: 20	8 (40%)	12 (60%)	
TN: 9	6 (66.7%)	3 (33.3%)	

**Table 2:** Factors significantly associated with residual tumour after re-excision for positive margins in the logistic regression analysis model.

FACTORS	ODDS RATIO	95% CI	*p* Value
N° positive margins	2.725885	1.6–4.69	<0.001
LVI	9.205266	2.39–35.5	0.001

## Data Availability

The data presented in this study are available on request from the corresponding author.
